# Direct auditory cortical input to the lateral periaqueductal gray controls sound-driven defensive behavior

**DOI:** 10.1371/journal.pbio.3000417

**Published:** 2019-08-30

**Authors:** Haitao Wang, Jiahui Chen, Xiaotong Xu, Wen-Jian Sun, Xi Chen, Fei Zhao, Min-Hua Luo, Chunhua Liu, Yiping Guo, Wen Xie, Hui Zhong, Tongjian Bai, Yanghua Tian, Yu Mao, Chonghuan Ye, Wenjuan Tao, Jie Li, Zahra Farzinpour, Juan Li, Jiang-Ning Zhou, Kai Wang, Jufang He, Lin Chen, Zhi Zhang

**Affiliations:** 1 Hefei National Laboratory for Physical Sciences at the Microscale, Chinese Academy of Sciences Key Laboratory of Brain Function and Disease, University of Science and Technology of China, Hefei, China; 2 Department of Biology and Chemistry, City University of Hong Kong, Kowloon, Hong Kong, China; 3 Wuhan Institute of Virology, Chinese Academy of Sciences, Wuhan, China; 4 Guangzhou Institutes of Biomedicine and Health, Chinese Academy of Sciences, Guangzhou, China; 5 Department of Psychology, Anhui Mental Health Center, Hefei, China; 6 Department of Neurology, the First Affiliated Hospital of Anhui Medical University, Hefei, China; 7 Department of Anesthesiology, the First Affiliated Hospital of Anhui Medical University, Hefei, China; New York University, UNITED STATES

## Abstract

Threatening sounds can elicit a series of defensive behavioral reactions in animals for survival, but the underlying neural substrates are not fully understood. Here, we demonstrate a previously unexplored neural pathway in mice that projects directly from the auditory cortex (ACx) to the lateral periaqueductal gray (lPAG) and controls noise-evoked defensive behaviors. Electrophysiological recordings showed that the lPAG could be excited by a loud noise that induced an escape-like behavior. *Trans*-synaptic viral tracing showed that a great number of glutamatergic neurons, rather than GABAergic neurons, in the lPAG were directly innervated by those in layer V of the ACx. Activation of this pathway by optogenetic manipulations produced a behavior in mice that mimicked the noise-evoked escape, whereas inhibition of the pathway reduced this behavior. Therefore, our newly identified descending pathway is a novel neural substrate for noise-evoked escape and is involved in controlling the threat-related behavior.

## Abstract 2

Threatening sounds can evoke a defensive behavior in animals to avoid potential harm. This study identifies a novel neural pathway in mice that projects directly from the auditory cortex to the lateral periaqueductal gray and controls defense-like behaviors evoked by a loud noise, supporting the notion that such behaviors are controlled by multiple neural circuits.

## Introduction

Mammals have evolved several strategies to deal with a dangerous situation that rely on behavioral responses such as freezing, escaping, and fighting [1,2], depending on the distance from where threat stimuli occur [3]. Sound is one of the natural threatening stimuli that elicit defensive behaviors. Different auditory stimuli elicit different behaviors, which may be harbored in distinct neural substrates [4]. For example, the superior colliculus (SC)-dorsolateral periaqueductal gray (dlPAG) circuit is recruited in frequency upsweeps–elicited escape [5], whereas the pontine reticular formation-ventrolateral tegmental nucleus circuit is recruited in loud white noise-induced startle responses [6]. Thus, different threat-related behaviors are controlled by a multiplicity of control systems [7,8]. Although many studies have explored the neural substrate of variously elicited defensive behaviors [9–15], the brain circuits underlying specific sound-driven behaviors are not fully understood.

Considerable evidence from chemical, electrical, and optogenetic studies has revealed an important role of the PAG in conditioned and innate behavioral reactions [5,16–20]. Some fear conditioning studies [9,21] have shown that disinhibition of the ventrolateral periaqueductal gray (vlPAG) results in a freezing reaction to a sound during threat conditioning. A previous study [22] has shown that activation of the dlPAG through stimulation of neural projections from the auditory cortex (ACx) in the cortex of the inferior colliculus (ICx) mediates a sound-evoked flight behavior. A recent study has shown that activation of excitatory inputs from the lateral hypothalamus to the PAG drives evasion [23]. Those studies indicate complex neural substrates and multiple controls of threat-related defensive behaviors centered around the PAG. These studies also implicate that there may exist some unknown neural circuits underlying these behaviors.

In the present study, using viral tracing strategies, we found that the ACx directly sends excitatory projections to glutamatergic neurons of the lateral periaqueductal gray (lPAG). Optical manipulations of the ACx→lPAG pathway recapitulated the behavioral phenotypes of mice exposed to threatening noise. Our study identifies a novel and cell type–specific glutamatergic (Glu)^ACx^→Glu^lPAG^ projection that controls noise-evoked escape.

## Results

### Involvement of Glu^lPAG^ neurons in noise-evoked escape behavior

**Fig 1:**
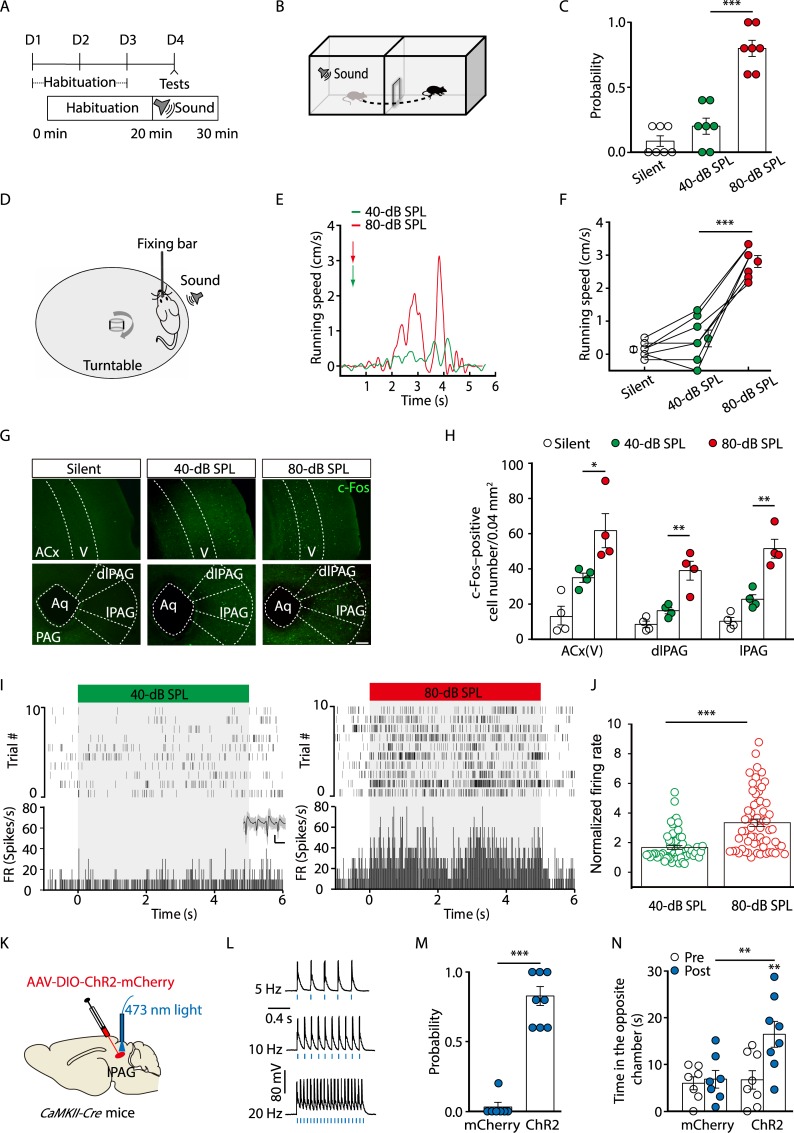
Involvement of the ACx and lPAG in noise-evoked defensive behaviors. (A) Time line of the behavioral protocol. (B) Schematic showing the sound-evoked escape behavior. (C) Probability of noise-evoked escape behavior (*t*_(6)_ = 6.874, *P* = 0.0005, paired *t* test, *n* = 7 mice). (D) Schematic showing how running speed is recorded in a head-fixed mouse. (E) Representative running speed traces. Arrowheads denote initiation of noise. (F) Running speed (*t*_(6)_ = 8.282, *P* = 0.0002, paired *t* test, *n* = 7 mice). (G) c-Fos expression pattern. The silent group was not exposed to noise. Scale bar, 100 μm. (H) Density of c-Fos–positive cells (ACx, *t*_(6)_ = 2.657, *P* = 0.0377; dlPAG, *t*_(6)_ = 4.049, *P* = 0.0067; lPAG, *t*_(6)_ = 4.793, *P* = 0.003. Unpaired *t* test, *n* = 4 slices from 3 mice/group). (I, J) Extracellular recordings for neuronal firing in the lPAG in response to 40-dB SPL (left panels) and 80-dB SPL (right panels) noise (I, top panel: raster plots; bottom panel: peristimulus time histograms; inset: spike waveforms) and summarized data of firing rates (J, *t*_(119)_ = 5.865, *P* < 0.0001, unpaired *t* test, *n* = 60–61 units from 3 mice). The firing rates are normalized to the baseline level for each neuron. Scale bar, 50 μV, 1 millisecond. (K) Schematic showing viral injection and light stimulation. (L) Sample traces of action potentials evoked by 473-nm light (blue bars) recorded from lPAG ChR2-positive neurons in acute midbrain slices. (M, N) Probability of light-evoked escape behavior (M, *U* = 0, *P* = 0.0003, Mann-Whitney *U* test, *n* = 7 or 8 mice) and time spent in the opposite chamber (N, *F*_(1, 13)_ = 5.94, *P* = 0.0299, two-way ANOVA, *n* = 7 or 8 mice). The underlying data for this figure can be found in S1 Data. Values are means ± SEM (*P < 0.05; **P < 0.01; ***P < 0.001). AAV, adeno-associated virus; ACx, auditory cortex; *CaMKII*, *Ca*^*2+*^*/calmodulin-dependent protein kinase II*; ChR2, channelrhodopsin-2; *Cre*, *cyclization recombination*; DIO, double-floxed inverted orientation; dlPAG, dorsolateral periaqueductal gray; FR, firing rate; lPAG, lateral periaqueductal gray; SPL, sound pressure level.

Previous work has shown that mice exhibit defensive behaviors in response to a loud sound [22,24]. We employed a similar paradigm to detect sound-evoked defensive responses. As shown in Fig 1A, the mice were allowed to habituate to an environment consisting of two chambers. Then, a sound was suddenly delivered in the chamber where the mouse was located. Following a loud white noise stimulus (80-dB sound pressure level [SPL], 5-s duration), the mouse immediately escaped toward the opposite chamber (Fig 1A–1C and S1 Video). To confirm this result, we trained head-fixed mice to freely run above a rotatable plate. Running speeds were measured with a rotatory encoder and recorded by a computer (Fig 1D). In response to the noise, the mice displayed immediate running (Fig 1E and 1F, S2 Video). In contrast, a low-level noise (30–40-dB SPL) with the same duration failed to induce escape behavior during the first trial (Fig 1E, and S1A Fig). The 80-dB SPL noise-evoked escape displayed clear adaptation from the fifth trial of sound stimulation (S1B–S1D Fig). Because the 80-dB SPL noise could reliably induce escape behavior without impairing hearing sensitivity [25], this stimulus was used for the rest of the study.

To reveal specific brain regions involved in the noise-elicited escape behavior, we assessed expression of the c-Fos protein in the brain following the noise stimulation [26,27]. Compared with control mice that were not exposed to the noise, mice subjected to the noise showed massive c-Fos expression in the limbic and auditory-related areas, including the ACx and lPAG (Fig 1G and 1H, S2 Fig). Furthermore, we performed extracellular recording in freely moving mice [28] and found that the noise at 80-dB SPL, rather than at 40-dB SPL, strongly increased neuronal firing rates in the lPAG (Fig 1I and 1J). In addition, we observed an adaption of noise-evoked neuronal firings in lPAG neurons but not in ACx neurons (S3 Fig). These results suggest that the excitability of lPAG neurons is required for the noise-evoked escape behavior.

Glu^lPAG^ neurons have been implicated in promoting flight behavior [9] and were activated by the escape-evoking noise. To test the role of Glu^lPAG^ neurons in noise-driven escape behavior, cyclization recombination (Cre)-dependent channelrhodopsin-2 (ChR2) virus (adeno-associated virus [AAV]- double-floxed inverted orientation [DIO]-ChR2-mCherry) was infused into the lPAG of *Ca*^*2+*^*/calmodulin-dependent protein kinase II* (*CaMKII*, an enzyme in glutamatergic neurons)*-Cre* mice to selectively activate Glu^lPAG^ neurons (Fig 1K). Whole-cell recordings from ChR2-expressing neurons showed that optical stimulation (473 nm, 10 milliseconds) of the lPAG reliably elicited action potential firing in acute brain slices (Fig 1L). After optical activation of Glu^lPAG^ neurons, mice showed multiple defensive behaviors in several paradigms that included escaping toward the opposite chamber, running on the rotatable plate, and wall rearing (Fig 1M and 1N, S4 Fig, S3 Video). Taken together, these results indicate that Glu^lPAG^ neurons are sufficient for noise-evoked defensive behavior.

### Dissection of the pathway from Glu^ACx^ to Glu^lPAG^

**Fig 2:**
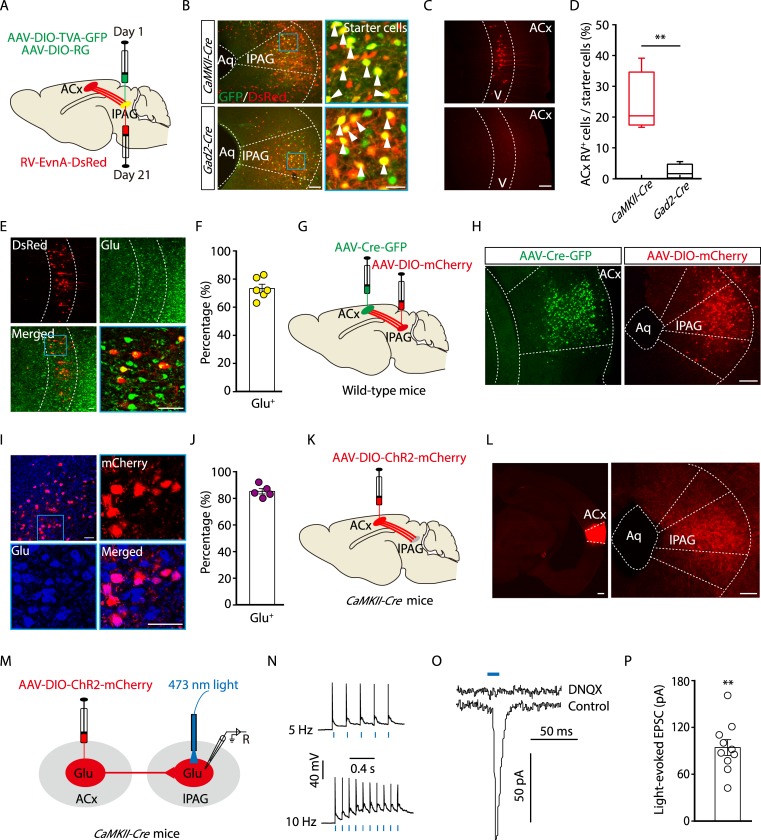
Dissection of the Glu^ACx^→Glu^lPAG^ pathway. (A) Schematic showing Cre-dependent retrograde *Trans*-monosynaptic RV tracing strategy. (B) Typical images of viral expression within the lPAG of *CaMKII-Cre* (top panels) and *Gad2-Cre* mice (bottom panels). Starter cells (yellow, arrowheads) co-expressing AAV-DIO-TVA-GFP, AAV-DIO-RVG (green), and RV-EnvA-ΔG-DsRed (red). Scale bars, 100 μm (left), 25 μm (right). (C) DsRed-labeled neurons in the ACx traced from Glu^lPAG^. Scale bar, 100 μm. (D) Quantification of DsRed-labeled ACx neurons (*t*_(6)_ = 4.21, *P* = 0.0056, unpaired *t* test, *n* = 4 slices from 4 mice/group). (E) DsRed signals co-localized with glutamate immunofluorescence in the ACx. Scale bar, 50 μm. (F) Percentage of DsRed-labeled neurons that contained glutamate in the ACx (*n* = 6 slices from 4 mice). (G) Schematic showing anterograde *Trans*-monosynaptic AAV tracing strategy. (H) Typical images of the ACx expressing AAV-Cre-GFP (left) and lPAG expressing AAV-DIO-mCherry (right) in wild-type mice. Scale bar, 100 μm. (I) mCherry signals indicative of Cre recombinase traced from the ACx co-localized with glutamate immunofluorescence in the lPAG. Scale bar, 50 μm. (J) Percentage of mCherry-labeled neurons that contained glutamate in the lPAG (*n* = 5 slices from 5 mice). (K) Schematic showing anterograde AAV tracing strategy for optogenetics. (L) Typical images of the ACx expressing AAV-DIO-ChR2-mCherry (left panel) and lPAG containing ChR2-expressing fibers from Glu^ACx^ (right panel). Scale bars, 100 μm. (M) Schematic showing ACx injection of AAV-DIO-ChR2-mCherry in *CaMKII-Cre* mice and recording configuration in acute midbrain slices. (N) Sample traces of action potentials evoked by 473-nm light (blue bars) recorded from ACx mCherry-positive neurons in acute cortical slices. (O, P) Typical light-evoked EPSCs (O) recorded from lPAG neurons after photostimulation of Glu^ACx^ terminals in the lPAG before and after bath application of 10 μM DNQX, and the summarized data (P) (*t*_(9)_ = 9.2479; *P <* 0.0001, one-sample *t* test, *n* = 10 cells). The underlying data for this figure can be found in S1 Data. Values are means ± SEM (**P < 0.01). AAV, adeno-associated virus; ACx, auditory cortex; Aq, aqueduct; *CaMKII*, *Ca*^*2+*^*/calmodulin-dependent protein kinase II*; ChR2, channelrhodopsin-2; *Cre*, *cyclization recombination*; DIO, double-floxed inverted orientation; DNQX, 6,7-dinitroquinoxaline-2,3(1H,4H)-dione; EnvA, avian sarcoma leucosis virus envelope protein; EPSC, excitatory postsynaptic current; *Gad2*, *glutamic acid decarboxylase 2*; GFP, green fluorescent protein; Glu, glutamatergic; lPAG, lateral periaqueductal gray; RV, rabies virus; RVG, rabies virus glycoprotein; TVA, the subgroup A avian leukosis virus receptor.

Next, we dissected the ACx→Glu^lPAG^ pathway. A retrograde *Trans*-monosynaptic tracing system was employed to characterize ACx→lPAG contacts. Cre-dependent adeno-associated helper viruses (AAV- elongation factor 1α [Ef1α]-DIO-the subgroup A avian leukosis virus receptor [TVA]-green fluorescent protein [GFP] and AAV-Ef1α-DIO-rabies virus glycoprotein [RVG]) were injected into the lPAG of *CaMKII-Cre* mice. After three weeks, rabies virus (RV) (avian sarcoma leucosis virus envelope protein [EnvA]-pseudotyped RV-ΔG-DsRed) was injected into the same site (Fig 2A and 2B). The presence of these helper viruses enabled the RV to spread retrogradely across monosynapses. Substantial populations of DsRed-labeled neurons were identified in multiple brain regions, including the ACx (Fig 2C and 2D, S5A and S5B Fig**)**. In the ACx, DsRed-positive cells with apical dendrites protruding towards layer I were distributed within layer V. The DsRed signal co-localized with the glutamate antibody (Fig 2E and 2F, S6F Fig). These results were confirmed with another retrograde tracer, cholera toxin subunit B (CTB)-555 (S6A–S6D Fig). In contrast, no DsRed signal was observed in the ACx from *glutamic acid decarboxylase 2* (*Gad2*, a GABA synthetic enzyme)*-Cre* mice using the same tracing system (Fig 2C and 2D, S5C and S5D Fig). These results indicate that Glu^lPAG^ neurons are selectively innervated by Glu^ACx^ neurons. To confirm this observation, a *Trans*-synaptic anterograde virus, AAV-Cre-GFP [24], was injected into the ACx of wild-type mice, and Cre-dependent AAV-DIO-mCherry was injected into the lPAG to visualize Cre recombinase-containing neurons (Fig 2G and 2H). We found that mCherry-positive cells concentrated mainly in the lPAG (Fig 2H) and appeared to be glutamatergic neurons (Fig 2I and 2J). In addition, AAV-DIO-eYFP was injected into the ACx of a reporter mouse strain (*CaMKII-Cre* × *Ai14-tdTomato* [*Ai14-tdTOM*]), which produced transgenic mice with red tdTomato-expressing glutamatergic neurons. We observed that two fluorophores, tdTOM, which indicates glutamatergic neurons, and enhanced yellow fluorescent protein (eYFP), which indicates ACx descending axon terminals, overlapped in the lPAG (S7D and S7E Fig).

To characterize functional connections of the Glu^ACx^→Glu^lPAG^ pathway, AAV-DIO-ChR2-mCherry was injected into the ACx of *CaMKII-Cre* mice (Fig 2K and 2L), and whole-cell patch-clamp recording was performed in acute brain slices (Fig 2M–2P). We observed mCherry-positive (glutamate) cell bodies in the ACx, and numerous mCherry-positive fibers projected to many brain regions, including the lPAG of *CaMKII-Cre* mice but not *Gad2-Cre* mice (Fig 2L and S7A–S7C Fig). At a holding potential of −70 mV, optical stimulation of ChR2-containing Glu^ACx^ terminals in the lPAG reliably elicited excitatory postsynaptic currents (EPSCs) in lPAG neurons, which could be blocked by the α-amino-3-hydroxy-5-methyl-4-isoxazolepropionic acid (AMPA) receptor antagonist, 6,7-dinitroquinoxaline-2,3(1H,4H)-dione (DNQX) (Fig 2O and 2P). Taken together, these data demonstrate that Glu^ACx^ neurons send monosynaptic projections to Glu^lPAG^ neurons.

### Activation of the Glu^ACx^→Glu^lPAG^ pathway evoked an escape behavior

**Fig 3:**
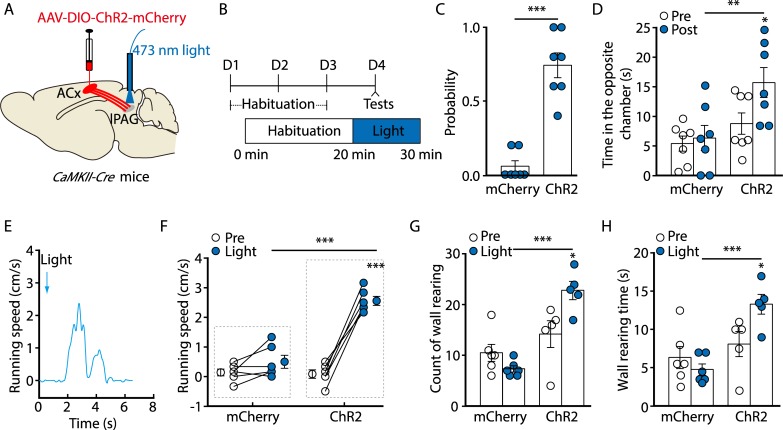
Optogenetic activation of the Glu^ACx^→Glu^lPAG^ pathway evoked defensive-like behaviors. (A) Schematic showing protocols for viral injection and light stimulation. (B) Time line of optogenetic experiments. (C, D) Probability of light-evoked escape behavior (C, *U* = 49, *P* = 0.0006, Mann-Whitney *U* test, *n* = 7 mice/group) and time spent in the opposite chamber (D, *F*_(1,12)_ = 7.22, *P* = 0.0198, *n* = 7 mice/group). (E, F) A representative trace (E) and speed of light-evoked running (F, *F*_(1,10)_ = 54.9, *P* < 0.0001, *n* = 6 mice/group). (G, H) Quantification of wall rearing events (G, *F*_(1,9)_ = 12.68, *P* = 0.0061, *n* = 5 or 6 mice) and wall rearing time (H, *F*_(1,9)_ = 8.04, *P* = 0.0196, *n* = 5 or 6 mice) before (pre) and during (light) light stimulation. The underlying data for this figure can be found in S1 Data. Values are means ± SEM (*P < 0.05; **P < 0.01; ***P < 0.001). Two-way ANOVA with Bonferroni post hoc analysis for (D), (F), (G), and (H). AAV, adeno-associated virus; ACx, auditory cortex; *CaMKII*, *Ca*^*2+*^*/calmodulin-dependent protein kinase II*; ChR2, channelrhodopsin-2; *Cre*, *cyclization recombination*; DIO, double-floxed inverted orientation; Glu, glutamatergic; lPAG, lateral periaqueductal gray.

Given increased excitability of lPAG neurons in the presence of noise, we investigated whether activation of the Glu^ACx^→Glu^lPAG^ pathway produces escape behaviors. As expected, after optical activation of ChR2-containing Glu^ACx^ terminals (5–8 mW) in the lPAG (Fig 3A and 3B), mice displayed a series of defensive behaviors, including escaping toward and spending more time in the opposite chamber, running on the rotatable plate, and wall rearing (Fig 3C–3H, S4 and S5 Videos). None of these behaviors was observed in the control group. In addition, escape behavior evoked by optical activation of the Glu^ACx^→Glu^lPAG^ pathway showed an adaptation similar to that evoked by noise (S8A and S8B Fig). Interestingly, co-application of subthreshold noise (40–50-dB SPL) and subthreshold light (4–5 mW) caused the mice to escape but failed to significantly change behavior when presented alone (S8C Fig). These results indicate that activation of the Glu^ACx^→Glu^lPAG^ pathway is sufficient to drive noise-evoked defensive behavior.

### Inhibition of the Glu^ACx^→Glu^lPAG^ pathway attenuated noise-evoked escape behavior

**Fig 4:**
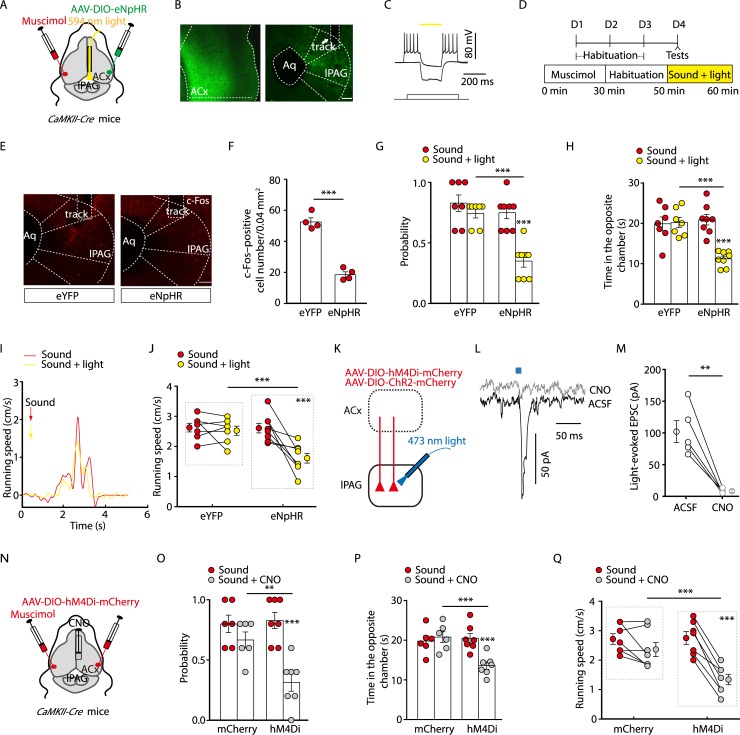
Inhibition of the Glu^ACx^→Glu^lPAG^ pathway reduced noise-evoked defensive behaviors. (A) Schematic showing protocols for optogenetic experiments. (B) Typical images of the ACx (left panel) expressing AAV-DIO-eNpHR3.0-eYFP and lPAG (right panel) containing eNpHR-expressing fibers from Glu^ACx^ with a track of an optical fiber (arrowhead). Scale bar, 100 μm. (C) Yellow light (594 nm) hyperpolarized an ACx neuron expressing eNpHR. (D) Time line of optogenetic experiments. (E) Noise-evoked c-Fos expression. Scale bar, 100 μm. (F) Density of c-Fos–positive cells (*t*_(6)_ = 10.58, *P* < 0.0001, unpaired *t* test, *n* = 4 slices from 4 mice/group). (G, H) Probability of noise-evoked escape behavior (G, *F*_(1, 13)_ = 10.23, *P* = 0.007, *n* = 7 or 8 mice) and time spent in the opposite chamber (H, *F*_(1, 13)_ = 16.74, *P* = 0.0013, *n* = 7 or 8 mice). (I, J) Representative recording traces (I) and summarized data (J, *F*_(1,13)_ = 8.46, *P* = 0.0122, *n* = 7 or 8 mice) of the speed of noise-evoked running in the absence and presence of light stimulation. (K) Schematic for verifying chemogenetic protocols. (L, M) Typical light-evoked EPSCs (L) recorded from lPAG neurons after photostimulation of ACx terminals in the lPAG before and after bath application of 10 μM CNO, and summarized data (M) (*t*_(4)_ = 5.76; *P* = 0.0045, paired *t* test, *n* = 5 cells). (N) Schematic showing protocols for chemogenetic experiments. (O, P) Probability of noise-evoked escape behavior (O, *F*_(1, 11)_ = 25.54, *P* = 0.0004, *n* = 6 or 7 mice), and time spent in the opposite chamber (P, *F*_(1,11)_ = 15.06, *P* = 0.0026, *n* = 6 or 7 mice). (Q) Summarized data (*F*_(1, 12)_ = 4.66, *P* = 0.0518, *n* = 7 mice/group) of the speed of noise-evoked running in the absence or presence of CNO. The underlying data for this figure can be found in S1 Data. Values are means ± SEM (**P < 0.01; ***P < 0.001). Two-way ANOVA with Bonferroni post hoc analysis for (G), (H), (J), (O), (P), and (Q). AAV, adeno-associated virus; ACSF, artificial cerebrospinal fluid; ACx, auditory cortex; Aq, aqueduct; *CaMKII*, *Ca*^*2+*^*/calmodulin-dependent protein kinase II*; ChR2, channelrhodopsin-2; CNO, clozapine-N-oxide; *Cre*, *cyclization recombination*; DIO, double-floxed inverted orientation; eNpHR, enhanced natronomonas pharaonis halorhodopsin; EPSC, excitatory postsynaptic current; eYFP, enhanced yellow fluorescent protein; Glu, glutamatergic; hM4Di, human Gi-coupled M4 muscarinic receptor; lPAG, lateral periaqueductal gray.

Next, we aimed to investigate whether inhibition of the Glu^ACx^→Glu^lPAG^ pathway prevents noise-evoked escape behavior. We infused a Cre-dependent AAV carrying enhanced natronomonas pharaonis halorhodopsin (eNpHR) (AAV-DIO-eNpHR3.0-eYFP) into the ACx to suppress activity of Glu^ACx^ axon projections in *CaMKII-Cre* mice (Fig 4A–4D). To avoid confounding effects from the contralateral cortex, we inactivated the other side of the ACx pharmacologically with muscimol [10] (Fig 4A and 4D). We found that, after noise stimulation, the number of c-Fos–positive cells was significantly lower in eNpHR-injected mice than in control mice after yellow light stimulation (Fig 4E and 4F). Optical inhibition of eNpHR-containing Glu^ACx^ terminals in the lPAG significantly reduced the probability of noise-evoked escape, time spent in the opposite chamber following the noise (Fig 4G and 4H), and speed of noise-evoked running for head-fixed mice (Fig 4I and 4J). The light stimuli did not change these behavioral responses at a statistical level in the control group (Fig 4G, 4H, and 4J). In addition, we found that the locomotion during light stimulation was not changed in the open-field test (S9 Fig).

We also used the chemogenetic method to silence ACx axon terminals in the lPAG by ACx infusion of AAV-DIO-human Gi-coupled M4 muscarinic receptor (hM4Di)-mCherry and lPAG infusion of clozapine-N-oxide (CNO). We found that light-evoked EPSCs in lPAG neurons through optically stimulating ChR2- and hM4Di-expressing ACx fibers were abolished by bath-applied CNO (Fig 4K–4M). Similar noise-evoked defensive behaviors were observed after chemogenetic inhibition of the ACx→lPAG pathway in *CaMKII-Cre* mice (Fig 4N–4Q). These results indicate that the Glu^ACx^→Glu^lPAG^ pathway is at least one of the underlying circuits that govern noise-evoked defensive behavior.

### Action of Glu^ACx^→Glu^lPAG^ pathway as a defense circuitry

**Fig 5:**
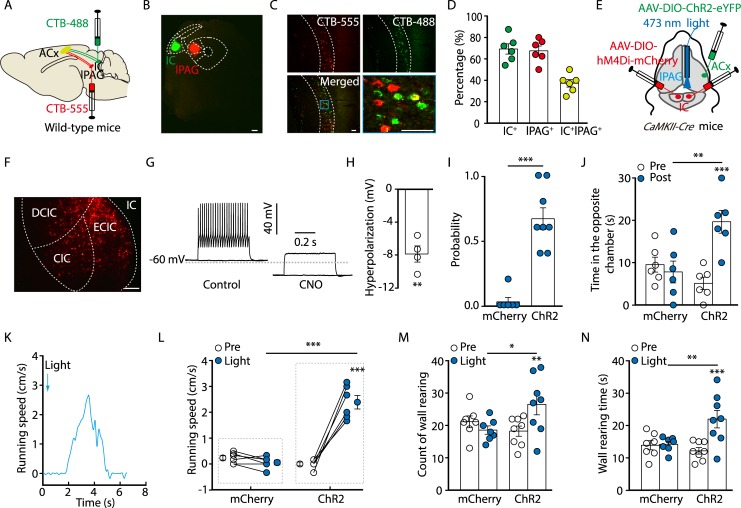
The Glu^ACx^→Glu^lPAG^ pathway mediated noise-evoked defensive behaviors independent of the ICx. (A) Schematic showing protocols for injections of CTB-555 and CTB-488 into the lPAG and ICx, respectively. (B) A fluorescent image showing tracer injection sites (ICx, green; lPAG, red). Scale bar, 100 μm. (C) Typical images of traced ACx neurons positive for CTB-555 and CTB-488 and a magnified view of the boxed region. Scale bar, 50 μm. (D) Percentage of neurons traced from the ICx (ICx-positive) and lPAG (lPAG-positive) out of CTB-positive neurons in layer V of the ACx. *n* = 6 slices from 3 mice. (E) Schematic showing protocols for silencing the ICx and the contralateral ACx, and optogenetic stimulation of the ACx terminals in the lPAG. (F) A typical image of the ICx expressing AAV-DIO-hM4Di-mCherry. Scale bar, 100 μm. (G, H) Perfusion of CNO (10 μM) hyperpolarized ICx neurons expressing hM4Di in acute slices (G) and summarized data (*H*, *t*_(3)_ = 7.96, *P* = 0.0041, one-sample *t* test, *n* = 4 cells). (I, J) Probability of light-evoked escape behavior (I, *U* = 0, *P* = 0.0007, Mann-Whitney *U* test, *n* = 6 or 8 mice) and time spent in the opposite chamber (J, *F*_(1, 10)_ = 14.53, *P* = 0.0034, *n* = 6 mice/group). (K, L) A representative trace (K) and summarized data (L, *F*_(1, 10)_ = 69.57, *P* < 0.0001, *n* = 6 mice/group) of the speed of light-evoked running before (pre) and during (light) light simulation. (M, N) Quantification of wall rearing events (M, *F*_(1, 13)_ = 13.49, *P* = 0.0028, *n* = 7 or 8 mice) and wall rearing time (N, *F*_(1, 13)_ = 11.58, *P* = 0.004, *n* = 7 or 8 mice). The underlying data for this figure can be found in S1 Data. Values are means ± SEM (*P < 0.05; **P < 0.01; ***P < 0.001). Two-way ANOVA with Bonferroni post hoc analysis for (J), (L), (M), and (N). AAV, adeno-associated virus; ACx, auditory cortex; *CaMKII*, *Ca*^*2+*^*/calmodulin-dependent protein kinase II*; ChR2, channelrhodopsin-2; CIC, central nucleus of the inferior colliculus; CNO, clozapine-N-oxide; *Cre*, *cyclization recombination*; CTB, cholera toxin subunit B; DCIC, dorsal cortex of the inferior colliculus; DIO, double-floxed inverted orientation; ECIC, external cortex of the inferior colliculus; Glu, glutamatergic; hM4Di, human Gi-coupled M4 muscarinic receptor; IC, inferior colliculus; ICx, cortex of the inferior colliculus; lPAG, lateral periaqueductal gray.

Previous work has shown that the ICx receives a descending projection from the ACx [22,29]. Thus, it is possible that action potentials can back-propagate to cell bodies of lPAG-projecting ACx neurons to activate ICx neurons when ACx→lPAG projection fibers are excited [30]. If this is the case, then behavioral effects following activation of the Glu^ACx^→Glu^lPAG^ pathway may result from sequential activation of the ICx→dlPAG pathway. To address this issue, we first co-injected respectively CTB-488 and CTB-555 into the lPAG and ICx to outline the ACx→lPAG and ACx→ICx pathways (Fig 5A and 5B). Seven days after the injection, we observed a minority of ACx neurons (37.16% ± 3.50%) that send collateral axons to both the lPAG and ICx (Fig 5C and 5D). These results are consistent with previous studies [22,24,31,32].

To determine whether behavioral responses evoked by activation of the ACx→lPAG pathway depend on the ICx, we used Cre-dependent expression of AAV-DIO-hM4Di-mCherry in the ICx and intraperitoneal injection of CNO to selectively inhibit ICx glutamatergic neurons in *CaMKII-Cre* mice (Fig 5E–5H). This is based on our finding that most retrogradely traced neurons from the lPAG were positive for glutamate in the ICx (S10A–S10C Fig). Three weeks after injection of AAV-DIO-hM4Di-mCherry into the ICx and that of AAV-DIO-ChR2-eYFP into the ACx, behavioral testing was conducted. We found that 50 minutes after CNO injection (3 mg/kg), optical activation of ChR2-containing Glu^ACx^ terminals in the lPAG still evoked escaping, running, and wall rearing (Fig 5I–5N). The role of ACx→lPAG projection in eliciting defensive reactions was corroborated in mice with ICx inactivation by AAV-human synapsin (hSyn)-hM4Di-mCherry (S11 Fig). In addition, after injection of AAV-eNpHR-eYFP into the ICx, we found most eNpHR-containing fibers in the dlPAG, rather than in the lPAG (S10D–S10F Fig). Optical inhibition of ICx terminals in the lPAG did not affect noise-evoked escape (S10G–S10I Fig).

**Fig 6:**
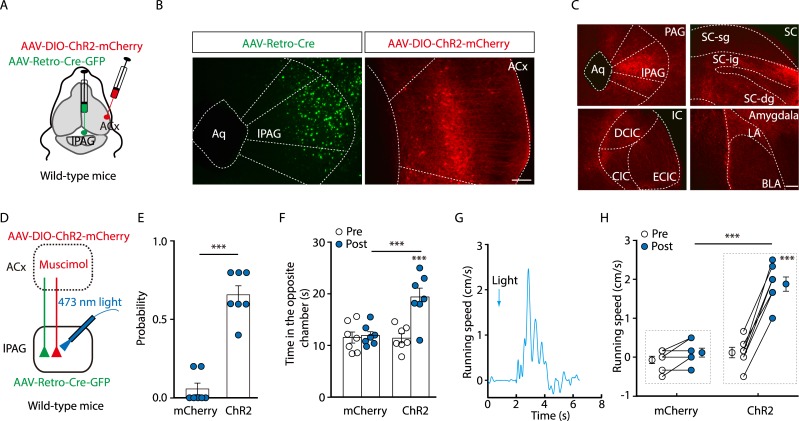
The Glu^ACx^→Glu^lPAG^ pathway mediated noise-evoked defensive behaviors independent of the collateral pathway. (A) Schematic showing a combinational viral strategy for mapping collateral pathways. (B) Typical images of the lPAG expressing AAV-Retro-Cre-GFP (left panel) and the ACx expressing AAV-DIO-ChR2-mCherry (right panel) in wild-type mice. Scale bar, 100 μm. (C) Typical images of collateral pathways of ACx→lPAG projection. Scale bar, 100 μm. (D) Schematic of viral injection, chemical inactivation, and light stimulation. (E, F) Probability of light-evoked escape behavior (E, *U* = 0, *P* = 0.0006, Mann-Whitney *U* test, *n* = 7 mice/group), and time spent in the opposite chamber (F, *F*_(1, 12)_ = 13, *P* = 0.0036, *n* = 7 mice/group). (G, H) A representative trace (G) and summarized data (H, *F*_(1, 12)_ = 32.03, *P* = 0.0001, *n* = 7 mice/group) of the speed of light-evoked running before (pre) and during (light) light simulation. The underlying data for this figure can be found in S1 Data. Values are means ± SEM (***P < 0.001). Two-way ANOVA with Bonferroni post hoc analysis for (F) and (H). AAV, adeno-associated virus; ACx, auditory cortex; Aq, aqueduct; BLA, basolateral amygdaloid nucleus; ChR2, channelrhodopsin-2; CIC, central nucleus of the inferior colliculus; Cre, cyclization recombination; DCIC, dorsal cortex of the inferior colliculus; DIO, double-floxed inverted orientation; ECIC, external cortex of the inferior colliculus; GFP, green fluorescent protein; Glu, glutamatergic; IC, inferior colliculus; LA, lateral amygdaloid nucleus; lPAG, lateral periaqueductal gray; SC, superior colliculus; SC-dg, deep gray layer of SC; SC-ig, intermediate gray layer of SC; SC-sg, superficial gray layer of SC.

To characterize the collateral pathway from lPAG-projecting ACx neurons, we employed a combinational viral strategy by lPAG infusion of AAV-Retro-Cre and ACx infusion of Cre-dependent AAV-DIO-ChR2, respectively (Fig 6A and 6B). The Cre recombinase-containing neurons were visible in the ACx and mCherry-containing projection fibers were observable in the lPAG, the inferior colliculus (IC), the medial SC (mSC), and the amygdala (Fig 6C). To rule out the involvement of these collateral pathways during optical stimulations, the ACx was silenced with muscimol before behavioral testing (Fig 6D). We found that the escape and running behaviors were reliably evoked by optical activation of ACx terminals in the lPAG after ACx inactivation (Fig 6E–6H). These results indicate that the Glu^ACx^→Glu^lPAG^ pathway functions as a defense circuit independent of the collateral pathway.

### Escape mediated by the mSC→dlPAG pathway

**Fig 7:**
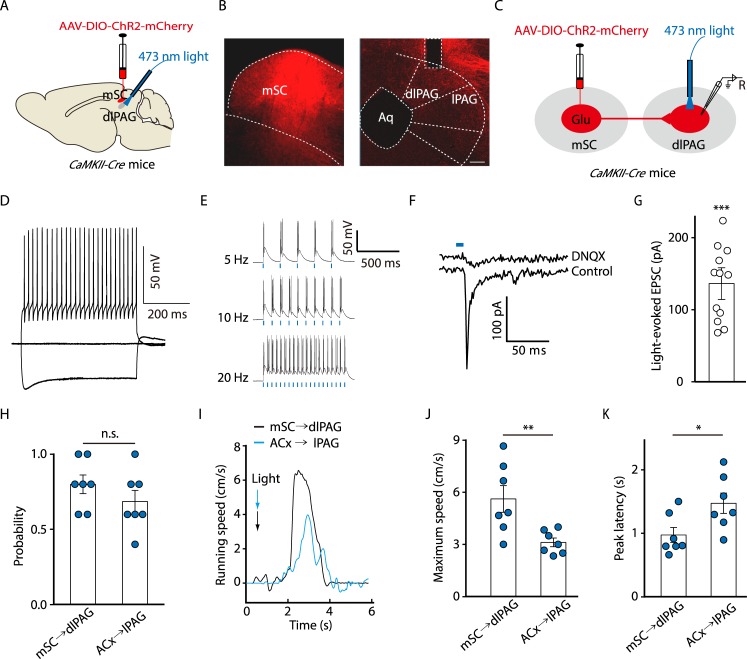
Escape mediated by the mSC→dlPAG pathway. (A) Schematic showing protocols for viral injection and light stimulation. (B) Typical images of the mSC (left panel) expressing AAV-DIO-ChR2-mCherry and dlPAG (right panel) containing mCherry-expressing fibers from mSC with a track of an optical fiber. Scale bar, 100 μm. (C) Schematic showing ACx injection of AAV-DIO-ChR2-mCherry in *CaMKII-Cre* mice and recording configuration in acute midbrain slices. (D) Sample traces of changes in membrane voltage in response to step current injections (−280, 0, 280 pA) in an mCherry-positive mSC neuron. (E) Sample traces of action potentials evoked by 473-nm light (blue bars) recorded from mSC mCherry-positive neurons. (F, G) Typical light-evoked EPSCs (F) recorded from dlPAG neurons after photostimulation of mSC terminals in the dlPAG before and after bath application of 10 μM DNQX, and summarized data (G) (*t*_(11)_ = 9.29; *P* < 0.001, one-sample *t* test, *n* = 12 cells). (H) Probability of light-evoked escape behavior by stimulating mSC→dlPAG and ACx→lPAG pathways, respectively (*t*_(12)_ = 1.188, *P* = 0.2577, unpaired *t* test, *n* = 7 mice/group). (I–K) Representative traces (I) and summarized data of the maximum speed (J, *t*_(12)_ = 3.085, *P* = 0.0094, unpaired *t* test,) and peak latency (K, *t*_(12)_ *=* 2.514, *P =* 0.0272, unpaired *t* test, *n* = 7 mice/group) of light-evoked running. The underlying data for this figure can be found in S1 Data. Values are means ± SEM (*P < 0.05; **P < 0.01; ***P < 0.001; n.s., not significant). AAV, adeno-associated virus; ACx, auditory cortex; Aq, aqueduct; *CaMKII*, *Ca*^*2+*^*/calmodulin-dependent protein kinase II*; ChR2, channelrhodopsin-2; *Cre*, *cyclization recombination*; DIO, double-floxed inverted orientation; dlPAG, dorsolateral periaqueductal gray; DNQX, 6,7-dinitroquinoxaline-2,3(1H,4H)-dione; Glu, glutamatergic; EPSC, excitatory postsynaptic current; lPAG, lateral periaqueductal gray; mSC, medial superior colliculus.

It has been reported that the mSC conveying cortical inputs to the dlPAG also mediates frequency upsweeps–evoked escape [5,24]. Our anterograde tracing experiment has shown that there were ACx fibers in the mSC (Fig 6C). In order to understand the role of this alternative pathway, we compared the escape behavior evoked by the optically activated ACx→lPAG pathway with that evoked by the optically activated mSC→dlPAG pathway. After infusion of AAV-DIO-ChR2-mCherry into the mSC, the fibers containing mCherry were visible in the dlPAG (Fig 7A–7C). The AAV-ChR2 protocol was verified by successful recordings of light-evoked action potentials in mSC neurons and EPSCs in dlPAG neurons (Fig 7D–7G). Upon optical activation of these terminals, the mice exhibited defensive behaviors, including escape toward the opposite chamber and running on the turntable (Fig 7H–7K). The running evoked by optical activation of the mSC→dlPAG pathway was faster in speed (Fig 7I and 7J) and shorter in the peak latency (Fig 7I and 7K) than that evoked by optical activation of the ACx→lPAG pathway. In addition, light-evoked EPSCs on dlPAG neurons by optical activation of mSC terminals were more pronounced than those associated with lPAG neurons subjected to optical activation of ACx terminals (Fig 7G and Fig 2P). We then examined whether the responses of these two circuits are dependent on the nature of auditory stimuli. Our results showed that escape behaviors evoked by noise were reduced when mSC→dlPAG projections were optically inhibited (S12C Fig). Similarly, escape behaviors evoked by frequency upsweeps were reduced when ACx→lPAG projections were optically inhibited (S12D Fig). These results indicate that the two circuits differentially contribute to the auditory-related defensive behaviors.

## Discussion

**Fig 8:**
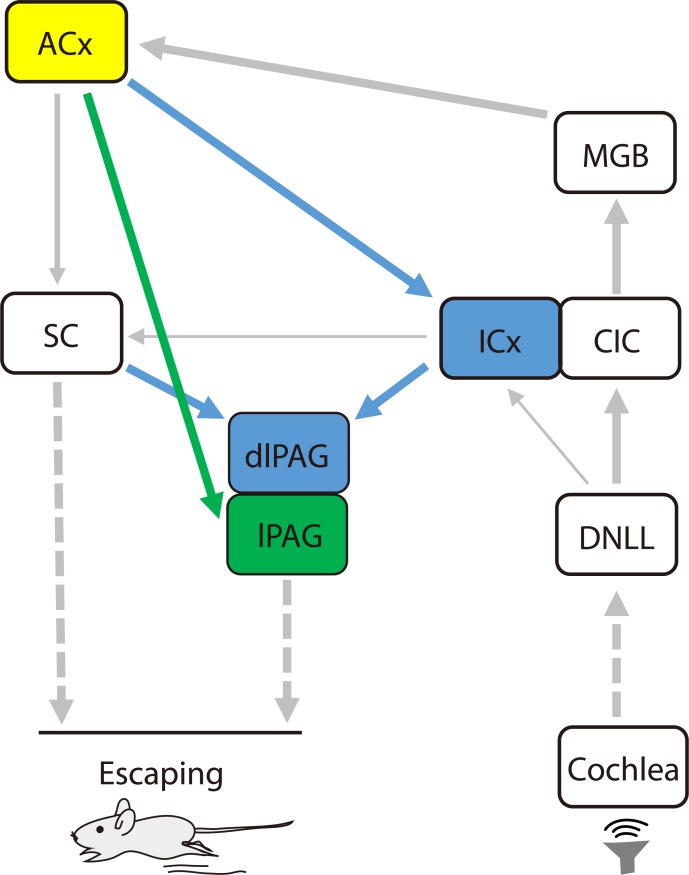
Proposed neural networks involved in noise-evoked defensive behaviors. Frightening sound activates Glu^ACx^ neurons, which send long-range excitatory projection onto Glu^lPAG^ neurons, the IC, and the SC. The activation of the lPAG (direct projection from ACx) or dlPAG (indirect projection from ACx with an intermediate of ICx or SC) leads to the generation of defensive behaviors. The possibility of the involvement of DNLL→ICx and IC→SC pathways also exists. ACx, auditory cortex; CIC, central nucleus of the inferior colliculus; dlPAG, dorsolateral periaqueductal gray; DNLL, the dorsal nucleus of the lateral lemniscus; Glu, glutamatergic; IC, inferior colliculus; ICx, dorsal and external cortex of the inferior colliculus; lPAG, lateral periaqueductal gray; MGB, medial geniculate body; SC, superior colliculus.

In this study, we have discovered a previously unexplored pathway for controlling noise-evoked escape behaviors that is cell specific and directly descending from the ACx to the lPAG (the Glu^ACx^→Glu^lPAG^ pathway). The robust supporting evidence for the existence of this pathway came from our viral tracing experiment and optogenetic experiment. Specifically, *Trans*-synaptic viral tracing revealed that a great number of glutamatergic neurons, rather than GABAergic neurons, in the lPAG are directly innervated by those in layer V of the ACx (Fig 2). Activation of this pathway by optogenetic manipulation mimicked the noise-evoked escape, whereas inhibition of the pathway reduced the escape (Fig 3 and Fig 4). Therefore, we have successfully identified a new pathway that is an important neural substrate for noise-evoked escape and is among the multiple neural circuits controlling threat-related behavior. Fig 8 shows proposed neural networks, including our newly identified one, that are involved in noise-evoked defensive behavior.

The PAG receives inputs from multiple regions, such as the amygdala, the hypothalamus, and the prefrontal cortex, which are involved in regulation of defensive behaviors [9,23,33,34]. However, our knowledge of the precise cell type–specific projections is limited. In this study, we found that glutamatergic neurons in the layer V of the ACx preferentially innervate lPAG glutamatergic neurons. This finding is interesting because glutamatergic neurons of both ACx and lPAG are flight promoting in auditory defensive behaviors [9,22].

As the output nucleus of defensive behavior for the PAG, each subdivision mediates a distinct behavior [17,18,23,27,35–37]. Specifically, sound-cued freezing behavior depends on the vlPAG [9], whereas sound-evoked escape largely relies on the dl/lPAG [22]. Our cell-specific viral tracing showed that layer V Glu^ACx^ neurons preferentially project to the lPAG but not the vlPAG. We also observed an adaptation of noise-evoked firings in the lPAG neurons but not in the ACx neurons, which might account for the adaption of noise-evoked escape behavior. These findings suggest a subregion-specific functional role of the lPAG in sound-driven defensive behaviors. Moreover, our extracellular recordings showed that noise stimulation strongly increased the neuronal firing rate in the lPAG. Given the spatial proximity of the lPAG to the dlPAG, it is necessary to discriminate lPAG and dlPAG neurons by the optotagging approach based on the input specificity in the future study.

Because corticofugal projections have been found to drive defensive behaviors [22,24], this raises the question of the nature of direct auditory cortical inputs to the lPAG, as well as their role in producing defensive behavior. Approximately 40% of ACx neurons send axon collateral inputs to both lPAG and ICx, resembling the projection pattern of the ventromedial hypothalamus to both the anterior hypothalamic nucleus and PAG [33]. In this study, we found that afferent inputs on PAG neurons differ greatly between these two pathways. ACx descending inputs preferentially target glutamatergic neurons, whereas ICx projecting fibers contact both glutamatergic and GABAergic neurons. Notably, optical activation of the Glu^ACx^→Glu^lPAG^ pathway elicited defensive behavior when the ICx was deactivated. These findings suggest that the newly identified Glu^ACx^→Glu^lPAG^ pathway controls noise-evoked escape by bypassing the ICx.

The mSC receives descending cortical inputs, and the mSC→dlPAG pathway contributes to sound-driven escape [5,24]. Whether the ACx→lPAG pathway functions differentially from the mSC→dlPAG pathway to trigger sound-elicited defensive behaviors requires further investigation. Nevertheless, we found that the running evoked by optical activation of the mSC→dlPAG pathway was faster in speed and shorter in the peak latency than that evoked by optical activation of the ACx→lPAG pathway. In addition, light-evoked EPSCs on dlPAG neurons by optical activation of mSC terminals were more pronounced than those associated with lPAG neurons subjected to optical activation of ACx terminals. These suggest the two circuits differentially contribute to the auditory-related defensive behaviors.

When acoustic information reaches the ACx, corticofugal projections send divergent inputs to lPAG neurons, to dlPAG-projecting ICx neurons, and to dlPAG-projecting mSC neurons (Fig 8). The activation of the PAG by monosynaptic ACx→lPAG projections may precede that by ACx→ICx (mSC)→dlPAG projections, suggesting that ACx→lPAG projections might rapidly mobilize the PAG, whereas the mSC and the ICx could amplify descending cortical controls [5,24].

Some fear conditioning studies showed that the ACx is indispensable for complex sound-cued fear but not for tone-cued fear [38–40]. Our present study also indicates an indispensable role of the ACx in the escape response to a white noise, but we are not sure whether the ACx is required for an escape response when a pure tone is used. It should be noted that there exist at least three major discrepancies between our behavioral paradigms and those in fear conditioning. (1) The innately elicited behaviors in the present study do not require, but learned fear behaviors do require, a training stage of associating a conditioned stimulus and unconditioned stimulus [41]. (2) The neural circuits recruited by these behaviors might also be quite different [2,4]. (3) We used a white noise for innately elicited escape, whereas others used complex sound, such as frequency-modulated sweep sound, for threat conditioning. Given those discrepancies, it is hard to predict how important the role of the ACx is in the escape behavior elicited by a complex sound. Further study is required to elucidate this issue.

The ACx evaluates the nature of threatening acoustic signals [22]. The cognitive control served by cortical processing could generate flexible defense behavior that is unlike the stereotyped behavior served by the subcortical circuit [42–45]. It has been reported that the ACx [46,47] and prefrontal cortex–zona incerta–lPAG circuit [20] may account for the extinction of learned fear. In this way, the neural network of the ACx might contribute to multiple auditory threat–related behaviors, such as noise-evoked escape behavior and its adaptation in the current study.

## Materials and methods

### Ethics statement

All animal protocols were approved by the Animal Care and Use Committee of the University of Science and Technology of China (USTCACUC1402021) and in accordance with the National Institutes of Health Guide for the Care and Use of Laboratory Animals.

### Animals

In all experiments, C57BL/6J, *CaMKII-Cre*, *Gad2-Cre*, and *Ai14* (RCL tdT) male mice (purchased from Charles River or Jackson Laboratories) at 8–10 weeks of age were used. Until the cannula surgery, the mice were housed five per cage in a colony with ad libitum access to water and food (standard mouse chow). They were maintained under a 12-hour light/dark cycle (lights on from 7:00 AM to 7:00 PM) at a stable temperature (23–25°C).

### Virus injection

Prior to surgery, the mice were fixed in a stereotactic frame (RWD, Shenzhen, China) under a combination of xylazine (10 mg/kg) anesthesia and ketamine (100 mg/kg) analgesia. A heating pad was used to maintain the core body temperature of the animals at 36°C. A volume of 100–300 nL virus (depending on the expression strength and viral titer) was injected using calibrated glass microelectrodes connected to an infusion pump (micro 4, WPI, Sarasota, FL) at a rate of 30 nL/minute. The coordinates were defined as dorsoventral (DV) from the brain surface, anterior-posterior (AP) from bregma, and mediolateral (ML) from the midline (in mm) [48,49].

For retrograde monosynaptic tracing, helper viruses that contained rAAV-Ef1α-DIO-RVG-WPRE-pA (AAV-DIO-RVG, AAV2/9, 2 × 10^12^ vg/mL) and rAAV-Ef1α-DIO-EGFP-2a-TVA-WPRE-pA (AAV-DIO-TVA-GFP, AAV2/9, 2 × 10^12^ vg/mL; 1:2, 200 nL) were co-injected into the lPAG of Cre transgenic mice (AP, 4.65 mm; ML, 0.6 mm; DV, 1.5 mm). After three weeks, RV-ENVA-ΔG-DsRed (2 × 10^8^ IFU/mL, 300 nL) was injected into the same site in the lPAG [50]. The retrograde tracers CTB-488 and CTB-555 (0.1 mg/mL, 100 nL, ThermoFisher) was also used to trace the ACx neurons projecting to the lPAG and ICx. Mice that had been anesthetized with pentobarbital (20 mg/kg, i.p.) were transcardially perfused 7 days after the last injection, and brain slices were prepared (40 μm) for DsRed tracing or co-staining with glutamate or GABA antibody.

For anterograde tracing, the Cre-dependent virus rAAV-Ef1α-DIO-hChR2(H134R)-mCherry-WPRE-pA (AAV-DIO-ChR2-mCherry, AAV2/9, 1.63 × 10^13^ vg/mL, 200 nL) was delivered into the ACx of Cre transgenic mice (AP, 2.45 mm; ML, 4.6 mm; DV, 0.9 mm). After four weeks, the expression of mCherry was detected in the whole brain. In some experiments, rAAV-Ef1α-DIO-eNpHR3.0-eYFP-WPRE-pA (AAV-DIO-eNpHR3.0-eYFP, AAV2/9, 1.18 × 10^13^ vg/mL) or AAV-hSyn-hChR2(H134R)-ER2-P2A-mCherry (AAV2/9, 5.7 × 10^12^ vg/mL, Taitool, Shanghai, China) was used in the ACx or mSC (AP, 4.6 mm; ML, 0.5 mm; DV, 0.9 mm) for optogenetic manipulation. For *Trans*-synaptic anterograde tracing, the AAV-CMV bGlobin-Cre-eGFP (AAV-Cre-GFP, AAV1, 2.79 × 10^13^ vg/mL, 250 nL, Taitool) was injected into the ACx, and the pAAV-Ef1α-DIO-mCherry-WPRE-pA into the lPAG at the same time in wild-type mice. The rAAV-Ef1α-DIO-hM4D(Gi)-mCherry-WPRE-pA (AAV-DIO-hM4Di-mCherry, AAV2/9, 3.69 × 10^13^ vg/mL) or AAV-hSyn-hM4D(Gi)-ER2-P2A-mCherry (AAV-hSyn-hM4Di-mCherry, AAV2/9, 5.66 × 10^12^ vg/mL, Taitool) viruses were delivered into the IC (AP, 5.01 mm; ML, 1.35 mm; DV, 0.9 mm) or ACx for chemogenetic manipulations three weeks after injection. In order to validate chemogenetic terminal inactivation, AAV-DIO-hM4Di-mCherry and AAV-DIO-ChR2-mCherry were co-injected into the ACx. In a viral strategy to visualize collateral pathways, AAV-Retro Plus-CMV-bGI-Cre-eGFP (AAV-Retro-Cre-GFP, AAV2/2Retro Plus, 2.03 × 10^13^ vg/mL, 70 nL, Taitool) was injected into the lPAG, and AAV-DIO-ChR2-mCherry into the ACx at the same time in wild-type mice. The rAAV-Ef1α-DIO-mCherry-WPRE-pA (AAV-DIO-mCherry, AAV2/8, 8.93 × 10^12^ vg/mL) and rAAV-DIO-eYFP-WPRE-pA (AAV-DIO-eYFP, AAV2/9, 1.95 × 10^12^ vg/mL) viruses were used as controls. Unless otherwise stated, all viruses were packaged by BrainVTA (Wuhan, China). All mice were *Trans*-cardially perfused with 0.9% saline, followed by ice-cold phosphate buffer (0.1 M) that contained 4% paraformaldehyde (PFA). Images of the signal expression were acquired with a confocal microscope (LSM 710, Carl Zeiss, Germany). Animals with missed injections were excluded.

### Optogenetic manipulations in vivo

An optical fiber was initially implanted into the lPAG, in the brain of an anesthetized mouse that had been immobilized in a stereotaxic apparatus. The implant was secured to the animal’s skull with dental cement. Chronically implantable fibers (diameter, 200 μm, Newdoon, Hangzhou) were connected to a laser generator using optic fiber sleeves. The delivery of blue light (473 nm, 5–8 mW, 20 Hz, 10-millisecond pulses) or yellow light (594 nm, 5–8 mW, constant) was controlled by a Master-8 pulse stimulator (A.M.P.I., Jerusalem, Israel). The same stimulus protocol was applied to the mice in the control group. The mice were allowed at least 10 days for recovery before injections to minimize stress during the behavioral assays. The location of the fibers was examined in all mice at the conclusion of the experiments, and data obtained from mice in which the fibers were located outside of the desired brain region were discarded. Behavioral assays were performed immediately after light stimulation.

### Local drug infusion

An internal stainless steel injector attached to a 10-μL syringe (Hamilton, Reno, NV) and an infusion pump was inserted into the guide cannula (I.D. 0.34 mm, RWD, Shenzhen, China) and used to infuse muscimol (0.2 μL, 0.5 mg/mL) into the left ACx or mSC [10] absent of virus injection or CNO (0.1 μL, 1 mg/mL) into the right lPAG at a flow rate of 100 nL per minute. The injector was slowly withdrawn 2 minutes after the infusion, and the behavioral assays were performed approximately 30 minutes after the infusion.

### In vivo electrophysiological recording

Animals were prepared for surgery as described above [28,51]. For chronic extracellular recordings (Fig 1I and 1J, S3 Fig), a custom-made four movable tetrode array was implanted into the lPAG (AP, 4.65 mm; ML, 0.6 mm; DV, 1.3 mm) and the ACx (AP, 2.45 mm; ML, 3.8 mm; DV, 0.9 mm). Each tetrode was made of four twisted fine platinum/iridium wires (12.5-μm diameter, California Fine Wire, Grover Beach, CA). The screw-based microdrive scaffolds for lowering the electrodes were cemented onto the skull. The mice were allowed to recover for at least 3 days before recordings were made. The recording sites were verified by passing an electrical current (20 μA, 15–20 seconds) to lesion the brain tissue at the end of all experiments. For head-fixed recordings (S11E and S11F Fig), a screw for head fixation was cemented on top of the skull. An array of two electrodes, one as recording electrode (approximately 1.0 MΩ, FHC, Bowdoin, ME) and the other tip-stripped electrode as reference, were positioned with a stepping-motor microdriver. Auditory stimuli were generated digitally using a computer-controlled Auditory Workstation from Tucker-Davis Technologies (TDT, Alachua, FL) and delivered through an open-field magnetic speaker (MF1, TDT) with an interval of 30 seconds. SPL was calibrated with a condenser microphone (Center Technology, Taiwan). Recording electrodes were attached to a 16-channel headstage, and neuronal signals were amplified, filtered at a bandwidth of 300–5,000 Hz, and stored using TDT software (OpenEX, TDT). Spike sorting was performed with a sorting method involving a T-Dis E-M algorithm built in Offline Sorter 4 (Plexon, USA). The firing rates of sorted units were calculated using Neuroexplorer 5 (Nex Technologies, USA). Peristimulus histograms (PSTHs) of firing rates were computed over a bin width of 10 milliseconds for each unit between −1 and 6 seconds, and in this time window the mean and SD of firing rates across all bins were calculated. The units with firing rates during noise stimulation between 99% confidence interval (mean ± 2.576 SD) are classified as not responsive ones, and those higher or lower than the confidence interval are sound-promoting or sound-inhibiting ones, respectively.

### Brain slice electrophysiology

#### Brain slice preparation

Acute brain slices were prepared as previously described [52]. Mice were deeply anesthetized with pentobarbital sodium (2% w/v, i.p.) and intracardially perfused with approximately 20 mL ice-cold oxygenated modified N-methyl-D-glucamine artificial cerebrospinal fluid (NMDG ACSF) that contained (in mM) 93 NMDG, 2.5 KCl, 1.2 NaH_2_PO_4_, 30 NaHCO_3_, 20 HEPES, 25 glucose, 2 thiourea, 5 Na-ascorbate, 3 Na-pyruvate, 0.5 CaCl_2_, 10 MgSO_4_, and 3 glutathione (GSH). The pH of the ACSF was 7.3–7.4, and osmolarity was 300–305 mOsm/kg. Coronal slices (300 μm) that contained the ACx, IC, mSC, or PAG were sectioned at 0.18 mm/second on a vibrating microtome (VT1200s, Leica, Germany). The brain slices were initially incubated in NMDG ACSF for 10–12 min at 33°C, followed by HEPES ACSF that contained (in mM) 92 NaCl, 2.5 KCl, 1.2 NaH_2_PO_4_, 30 NaHCO_3_, 20 HEPES, 25 glucose, 2 thiourea, 5 Na-ascorbate, 3 Na-pyruvate, 2 CaCl_2_, 2 MgSO_4_, and 3 GSH (pH, 7.3–7.4; osmolarity, 300–305 mOsm/kg) for at least 1 hour at 25°C. The brain slices were transferred to a slice chamber (Warner Instruments, Hamden, CT) for electrophysiological recording and were continuously perfused with standard ACSF that contained (in mM) 124 NaCl, 2.4 CaCl_2_, 5 KCl, 1.3 MgSO_4_, 26.2 NaHCO_3_, 1.2 KH_2_PO_4_, and 10 glucose (pH, 7.3–7.4; osmolarity, 300–305 mOsm/kg) at 2.5–3 mL/minute at 32°C. The temperature of the ACSF was maintained by an in-line solution heater (TC-344B, Warner Instruments).

#### Whole-cell patch-clamp recordings

Neurons in the slice were visualized using a 40× water-immersion objective on an upright microscope (BX51WI, Olympus, Japan) equipped with interference contrast (IR/DIC) and an infrared camera connected to the video monitor. Whole-cell patch-clamp recordings were obtained from visually identified ACx layer V, ICx, mSC, or PAG cells. Patch pipettes (3–5 MΩ) were pulled from borosilicate glass capillaries (VitalSense Scientific Instruments Co., Wuhan, China) with an outer diameter of 1.5 mm on a four-stage horizontal puller (P1000, Sutter Instruments, Novato, CA) and filled with intracellular solution that contained (in mM) 130 K-gluconate, 2 MgCl_2_, 5 KCl, 0.6 EGTA, 10 HEPES, 2 Mg-ATP, 0.3 Na-GTP (pH, 7.3–7.2; osmolarity, 285–290 mOsm/kg). The neurons were held at −70 mV in voltage-clamp mode to record the membrane currents and at 0 pA in current-clamp mode to record the membrane voltages. The signals were acquired via a Multiclamp 700B amplifier (Molecular Devices, Sunnyvale, CA), low-pass filtered at 2.8 kHz, digitized at 10 kHz, and analyzed with Clampfit 10.7 software (Molecular Devices). If the series resistance changed more than 20% during the recording, the experimental recording was immediately terminated.

#### Light-evoked responses

Optical stimulation was delivered using a laser (Shanghai Fiblaser Technology Co., China) through an optical fiber 200 μm in diameter positioned 0.2 mm from the surface of the brain slice. To test the functional characteristics of AAV-DIO-ChR2, fluorescently labeled neurons expressing ChR2 in *CaMKII-Cre* mice 3–4 weeks after virus injection were visualized and stimulated with a blue light (473 nm, 5–10 mW) using 5-Hz, 10-Hz, or 20-Hz stimulation protocols with a pulse width of 10 milliseconds. Similarly, the function of AAV-DIO-eNpHR3.0 was assessed in fluorescently labeled neurons expressing eNpHR by applying sustained yellow light stimulation (594 nm, 5–10 mW, 200 milliseconds). For electrophysiological recording of monosynaptic postsynaptic currents, 1 μM tetrodotoxin (TTX) and 1 mM 4-aminopyridine (4-AP) were added to the bath solution to eliminate the polysynaptic components, and blue light (473 nm, 10-millisecond pulse) was delivered to the lPAG or dlPAG of *CaMKII-Cre* mice in which the ACx or mSC had been injected with AAV-DIO-ChR2-mCherry. DNQX (10 μM) was used to block glutamate receptors. Unless otherwise stated, all drugs were purchased from Sigma-Aldrich (St. Louis, MO). TTX was obtained from Hebei Aquatic Science and Technology Development Company, China.

### Immunohistochemistry

The mice were deeply anesthetized with pentobarbital sodium (50 mg/kg, i.p.) and sequentially perfused with saline and 4% (w/v) PFA. The brains were subsequently removed and postfixed in 4% PFA at 4°C overnight. After cryoprotection of the brains with 30% (w/v) sucrose, coronal sections (40 μm) were cut on a cryostat (Leica CM1860, Germany) and used for immunofluorescence. The sections were incubated in 0.3% (v/v) Triton X-100 for 0.5 hour, blocked with 10% donkey serum for 1 hour at room temperature, and incubated with primary antibodies, including anti-c-Fos (1:500, rabbit, Santa Cruz Biotechnology, Dallas, TX), anti-glutamate (1:500, rabbit, Sigma-Aldrich), and anti-GABA (1:500, rabbit, Sigma Aldrich) at 4°C for 24 hours, followed by the corresponding fluorophore conjugated secondary antibodies for 2 hours at room temperature. Fluorescence signals were visualized using Leica DM2500 and Zeiss LSM710 microscopes and analyzed using ImageJ 1.4 (NIH). For counting immunoreactive cells, the 8-bit grayscale image was background subtracted before applying a threshold to all images. The threshold was adjusted within 10% of the average intensity, and cells at or above the threshold are considered immunopositive.

### Behaviors

All behavioral tests were conducted within a soundproof chamber, and mice were habituated for 3 days prior to testing. Auditory stimuli of white noise or frequency upsweeps (frequency-modulated upsweep from 17 to 20 kHz over 3 seconds) were generated through a RZ6 Multi I/O Processor (TDT, Alachua, FL) and delivered by MF1 open-field magnetic speakers. SPL was calibrated carefully. During each testing session, behavior was recorded using an infrared camera. Blue light was generally delivered at 20 Hz for 10–20 seconds with the exception of 5 minutes for testing wall rearing. The duration of the yellow light was identical to that of sound. The experimental area was cleaned with 75% ethanol after each test to remove olfactory cues from the apparatus. To avoid behavioral adaptation, the mouse for the optogenetic experiment (Fig 3, Fig 5, Fig 6, and Fig 7) was not exposed to sound unless the synergetic effect of light and noise on escape behavior was evaluated (S8 Fig).

#### Escape behavior test

Mice were placed in a behavioral box (40 × 25 × 25 cm) consisting of two chambers and a middle plate. Two speakers were placed on the walls of each chamber. Each mouse was allowed to freely explore the surroundings and cross the opening in the plate toward the opposite side. Sound was delivered from the chamber where the mouse was located. Time spent in the opposite chamber was determined within 30 seconds from the end of sound or light delivery. The probability and time spent in the opposite chamber for each mouse were averaged across five trials.

#### Running behavior test for head-fixed mice

Mice with implanted optical fibers were used in this set of experiments. Each mouse was clamped to a fixing bar on the optic fiber sleeve and allowed to adapt to head fixation. Then, the mouse could run freely on a Plexiglass circular plate (diameter, 30 cm) that was connected to a rotatory encoder used to record running speed. The data were digitized and stored on the computer for offline analysis. Sounds were delivered from a speaker placed 10 cm from the ear of each head-fixed mouse.

#### Wall rearing behavior test

Each mouse was placed in a single box (50 × 50 × 60 cm) to observe blue light–evoked wall rearing behavior. Offline inspection of the video was performed to determine the number and total duration of wall rearing within a time window of 5 minutes before and during light stimulation.

#### Open-field test

Mice were placed in one corner of an open-field apparatus that consisted of a square area (25 × 25 cm) and a marginal area (50 × 50 × 60 cm); the mice were allowed to freely explore their surroundings. The animals’ movement trajectories were recorded for 5 minutes using EthoVision XT software 8.5 (Noldus Information Technology).

### Statistical analysis

OriginPro 2017 (OriginLab Corporation) or GraphPad Prism 7 (GraphPad Software) were used for statistical analysis and graphing. All values are expressed as the means ± SEM. Student *t* test and two-way repeated measures ANOVA were used to evaluate statistical significance level unless otherwise stated. Mann-Whitney *U* test was used if data are not normally distributed. Significance levels are indicated as *P < 0.05, **P < 0.01, and ***P < 0.001.

## Supporting information

S1 Data Data used to generate the figures. (XLSX)

S1 Fig Adaptation of noise-evoked escape behavior. (A) Probability of escape evoked by noise with different SPL during the first trial (*n* = 8 mice). (B) Probability of sound-evoked escape (noise, 80-dB SPL) over 10 consecutive trials at an interval of 60 seconds (*n* = 8 mice). (C) Escape probability averaged across the first five trials (trials 1–5) and second five trials (trials 6–10) (*U* = 49, *P* = 0.0006, Mann-Whitney *U* test, *n* = 7 mice). (D) Speed of sound-evoked running averaged over the first five trials (*n* = 7 mice). The underlying data for this figure can be found in S1 Data. Values are means ± SEM (***P < 0.001). SPL, sound pressure level.
(EPS)

S2 Fig Expression of c-Fos. (A) c-Fos–positive cells in the IC, MGB, and amygdala in the indicated groups. Scale bar, 100 μm. (B) Number of c-Fos–positive cells per 0.04 mm^2^ imaging area in the ACx, lPAG, IC, MGB, and amygdala (ACx, *t*_(6)_ = 4.35, *P* = 0.0048; lPAG, *t*_(6)_ = 7.09, *P* = 0.0004; ICx, *t*_(6)_ = 8.31, *P* = 0.0002; MGB, *t*_(6)_ = 2.71; *P* = 0.0349; BLA, *t*_(6)_ = 5.40, *P* = 0.0017; CeA, *t*_(6)_ = 0.72, *P* = 0.4962. Unpaired *t* test, *n* = 4 slices from 3 mice). (C) c-Fos–positive cells co-localized mostly with glutamate immunofluorescence in the lPAG. Scale bar, 25 μm. (D) Few c-Fos–positive cells co-localized with GABA immunofluorescence in the lPAG. Scale bar, 25 μm. (E) Percentage of c-Fos–positive cells that contained glutamate or GABA in the lPAG (*n* = 6 slices from 3 mice). The underlying data for this figure can be found in S1 Data. Values are means ± SEM (*P < 0.05; **P < 0.01; ***P < 0.001; n.s., not significant). ACx, auditory cortex; BLA, basolateral amygdaloid nucleus; CeA, central nucleus of the amygdala; CeC, capsular division of the central amygdaloid nucleus; CeL, lateral division of central amygdaloid nucleus; CeM, medial division of the central amygdaloid nucleus; CIC, central nucleus of the inferior colliculus; DCIC, dorsal cortex of the inferior colliculus; ECIC, external cortex of the inferior colliculus; IC, inferior colliculus; ICx, cortex of the inferior colliculus; LA, lateral amygdaloid nucleus; lPAG, lateral periaqueductal gray; MGB, medial geniculate body; MGD, dorsal part of the medial geniculate nucleus; MGM, medial part of the medial geniculate nucleus; MGV, ventral part of the medial geniculate nucleus.
(EPS)

S3 Fig In vivo electrophysiology. (A) Extracellular recordings in the vlPAG of freely moving mice. (B) Sample voltage trace recorded in the lPAG. (C) Histological verification of the recording site (triangle). (D) Percentage of units in the lPAG responding to 80-dB SPL noise. (E) Extracellular recordings for neuronal firing in the ACx in response to 80-dB SPL noise (top panel, raster plots; bottom panel, peristimulus time histograms; inset, spike waveforms). Scale bar, 50 μV, 1 millisecond. (F) The normalized firing rates over 10 consecutive trials at an interval of 30 seconds in the lPAG neurons and ACx neurons. (G) The normalized noise-evoked firing rates averaged across the first five trials (trials 1–5) and second five trials (trials 6–10) in the lPAG (left panel, *t*_(20)_ = 3.67, *P* = 0.0015, paired *t* test, *n* = 21 from 3 mice) and ACx (right panel, *t*_(19)_ = 0.92, *P* = 0.369, paired *t* test, *n* = 20 from 2 mice). The underlying data for this figure can be found in S1 Data. Values are means ± SEM (**P < 0.01; n.s., not significant). ACx, auditory cortex; FR, firing rate; lPAG, lateral periaqueductal gray; SPL, sound pressure level; vlPAG, ventrolateral periaqueductal gray.
(EPS)

S4 Fig Optical activation of Glul^PAG^ neurons evoked defensive behaviors. (A) Left panel: schematic showing viral injection, laser stimulation, and recording configuration in acute slices. Right panel: typical image of the lPAG expressing AAV-DIO-ChR2-mCherry. Scale bar, 100 μm. (B, C) Sample traces of changes in membrane voltage (B) in response to step current injections (−200, −150, −100, −50, 0, and 200 pA) and membrane currents (C) in response to light stimuli at 5, 10, and 20 Hz in an lPAG neuron expressing ChR2. (D, E) A representative trace (D) and summarized data (E) of the speed of light-evoked running before (pre) and during (light) light stimulation (*F*_(1, 10)_ = 191.2, *P* < 0.0001, *n* = 6 mice/group). (F–H) Quantification of wall rearing events (F, *F*_(1, 8)_ = 22.53, *P* = 0.0015, *n* = 5 mice/group), wall rearing time (G, *F*_(1, 8)_ = 21.38, *P* = 0.0017, *n* = 5 mice/group), and jumping events (H, *F*_(1, 8)_ = 8.467, *P* = 0.0196, *n* = 5 mice/group) before (pre) and during (light) light stimulation. The underlying data for this figure can be found in S1 Data. Values are means ± SEM (**P < 0.01; ***P < 0.001). Two-way ANOVA with Bonferroni post hoc analysis for (E), (F), (G), and (H). AAV, adeno-associated virus; ChR2, channelrhodopsin-2; DIO, double-floxed inverted orientation; Glu, glutamatergic; lPAG, lateral periaqueductal gray.
(EPS)

S5 Fig Mapping presynaptic inputs onto glutamatergic and GABAergic neurons in the lPAG. (A) Schematic showing the Cre-dependent retrograde *Trans*-monosynaptic RV tracing strategy in *CaMKII-Cre* mice. (B) Typical images of retrogradely traced DsRed-positive neurons in the IC (B1), the cingulate cortex (Cg, B2), the lateral hypothalamus (LH, B3), the medial hypothalamus (MH, B4), and the somatosensory cortex (S1, B5). (C) Schematic showing the Cre-dependent retrograde *Trans*-monosynaptic RV tracing strategy in *Gad2-Cre* mice. (D) Typical images of retrogradely traced DsRed-positive neurons in the IC (D1), Cg (D2), LH (D3), MH (D4), and S1 (D5). Scale bars, 100 μm. *CaMKII*, *Ca*^*2+*^*/calmodulin-dependent protein kinase II*; CA1, hippocampus CA1 field; Cg1, cingulate cortex, area 1; Cg2, cingulate cortex, area 2; *Cre*, *cyclization recombination*; DM, dorsomedial hypothalamic nucleus; f, fornix; *Gad2*, *glutamic acid decarboxylase 2*; IC, inferior colliculus; LH, lateral hypothalamus; LMol, lacunosum moleculare layer of the hippocampus, lPAG, lateral periaqueductal gray; MCLH, magnocellular nucleus of the lateral hypothalamus; MH, medial hypothalamus; Or, oriens layer of the hippocampus; PeF, perifornical nucleus; Py, pyramidal cell layer of the hippocampus; RV, rabies virus; S1, somatosensory cortex; S1BF, barrel field of the primary somatosensory cortex; S1Tr, trunk region of the primary somatosensory cortex; VMHC, central part of the ventromedial hypothalamic nucleus; VMHDM, dorsomedial part of the ventromedial hypothalamic nucleus; VMHVL, ventrolateral part of the ventromedial hypothalamic nucleus; 3V, third ventricle.
(EPS)

S6 Fig Cell type identification of ACx neurons projecting to the lPAG. (A) Schematic showing the retrograde CTB-555 tracing strategy. (B) Typical images of the injection site in the lPAG (left panel) and retrogradely traced CTB-positive neurons in the ACx (right panel). Scale bars, 50 μm. (C) CTB signals in the ACx co-localized with glutamate immunofluorescence. Scale bars, 50 μm. (D) Percentage of glutamate-positive neurons out of CTB-positive ACx cells (*n* = 6 slices from 3 mice). (E) Schematic showing the Cre-dependent retrograde *Trans*-monosynaptic RV tracing strategy in *CaMKII-Cre* mice. (F) ACx DsRed signals traced from the lPAG did not overlap with GABA immunofluorescence. Scale bar, 50 μm. The underlying data for this figure can be found in S1 Data. Values are means ± SEM. ACx, auditory cortex; *CaMKII*, *Ca*^*2+*^*/calmodulin-dependent protein kinase II*; *Cre*, *cyclization recombination*; CTB, cholera toxin subunit B; lPAG, lateral periaqueductal gray; RV, rabies virus.
(EPS)

S7 Fig Mapping output of ACx neurons. (A) Schematic showing the Cre-dependent anterograde AAV tracing strategy. (B, C) Typical images of the ACx expressing AAV-DIO-ChR2-mCherry and projecting fibers in the lPAG, IC, MGB, and amygdala in *CaMKII-Cre* (top panels) and *Gad2-Cre* mice (bottom panels). Scale bar, 100 μM. (D) Schematic showing the Cre-dependent anterograde AAV tracing strategy in *CaMKII-Cre × Ai14-tdTOM* mice. (E) Typical images of the ACx (left top) and lPAG (left bottom), and a magnified view of the boxed region (right panels). Scale bars, 100 μm (left), 25 μm (right). AAV, adeno-associated virus; ACx, auditory cortex; *CaMKII*, *Ca*^*2+*^*/calmodulin-dependent protein kinase II*; ChR2, channelrhodopsin-2; *Cre*, *cyclization recombination*; DIO, double-floxed inverted orientation; *Gad2*, *glutamic acid decarboxylase 2*; IC, inferior colliculus; lPAG, lateral periaqueductal gray; MGB, medial geniculate body; *tdTOM*, tdTomato.
(EPS)

S8 Fig Synergetic effect of sound and light stimuli on escape behavior. (A) Probability of light-evoked escape behavior. (B) Probability of light-evoked escape averaged across the first five trials (trials 1–5) and second five trials (trials 6–10) (*U* = 43.5, *P* = 0.0169, Mann-Whitney *U* test, *n* = 7 mice). (C) Co-application of subthreshold sound (SS, 40–50-dB SPL) and subthreshold light (SL, 4–5 mW) caused the mice to escape but failed to significantly change behavior when presented alone (*n* = 8 mice). The underlying data for this figure can be found in S1 Data. Values are means ± SEM (*P < 0.05). SL, subthreshold light; SPL, sound pressure level; SS, subthreshold sound.
(EPS)

S9 Fig eNpHR-based optogenetic manipulations did not change locomotor behavior. (A–C) The moving trajectory (A) of mice with eNpHR-based optogenetic manipulation in the absence and presence of yellow light, and the summarized data of the travel distances (B, *F*_(1, 12)_ = 0.1466, *P* = 0.7085, *n* = 7 mice/group) and moving velocity (C, *F*_(1, 12)_ = 0.2906, *P* = 0.5997, *n* = 7 mice/group). (D) Optogenetic inhibition reduced noise-evoked escape (*t*_(6)_ = 13, *P* < 0.0001, paired *t* test, *n* = 7 mice/group). The underlying data for this figure can be found in S1 Data. Values are means ± SEM (***P < 0.001; n.s., not significant). Two-way ANOVA with Bonferroni post hoc analysis for (B) and (C). eNpHR, enhanced natronomonas pharaonis halorhodopsin.
(EPS)

S10 Fig ICx→PAG projections. (A) Schematic showing the Cre-dependent retrograde RV tracing strategy. (B) DsRed signals traced from the lPAG were co-localized with the glutamate immunofluorescence in the ICx. Scale bars, 50 μm. (C) Percentage of Glu-positive neurons out of DsRed positive ICx cells (*n* = 5 slices from 4 mice). (D) Schematic showing viral injection. (E, F) Typical images of the ICx (E) and the PAG (F) expressing AAV-DIO-eNpHR3.0-eYFP. Scale bar, 100 μm. (G) Schematic showing viral injection, chemical inactivation, and light stimulation. (H, I) Probability of noise-evoked escape behavior (H, *t*_(12)_ = 1.179, *P* = 0.2614, unpaired *t* test, *n* = 7 mice/group) and running speed of noise-evoked running (I, *F*_(1,12)_ = 0.0131, *P* = 0.9107, two-way ANOVA, *n* = 7 mice/group) in mice with optically inhibited ICx→lPAG projection. The underlying data for this figure can be found in S1 Data. Values are means ± SEM (n.s., not significant). AAV, adeno-associated virus; CnF, cuneiform nucleus; Cre, cyclization recombination; DIO, double-floxed inverted orientation; eNpHR, enhanced natronomonas pharaonis halorhodopsin; eYFP, enhanced yellow fluorescent protein; Glu, glutamatergic; ICx, cortex of the inferior colliculus; lPAG, lateral periaqueductal gray; RV, rabies virus.
(EPS)

S11 Fig Inactivation of the IC neurons with chemogenetics. (A) Schematic showing chemogenetic experiments. (B) A typical image of the IC expressing AAV-hSyn-hM4Di-mCherry in wild-type mice. (C) c-Fos expression induced by noise following systemic injection of CNO. Scale bar, 200 μm. (D) Number of c-Fos–positive cells per 0.04-mm^2^ imaging area (*t*_(5)_ = 10.30, *P* < 0.0001, unpaired *t* test, *n* = 6 slices from 3 mice). (E, F) Multi-unit recordings in the ICx of head-fixed mice in vivo (top panel, raster plots; bottom panel, peristimulus time histograms) (E) before (left panel) and after (right panel) CNO injection, and summarized data (F, Saline, *t*_(8)_ = 2.04, *P* = 0.0755; CNO, *t*_(13)_ = 4.52, *P* = 0.0006. Paired *t* test, *n* = 9 or 14 neurons). (G, H) Summarized data of the probability (G, *CaMKII-Cre*, *U* = 0, *P* = 0.0022, *n* = 6 mice/group; wild-type, *U* = 0, *P* = 0.0002, *n* = 7 or 8 mice. Unpaired *t* test) and time spent in the opposite chamber (H, *CaMKII-Cre*, *F*_(1, 10)_ = 114.7, *P* < 0.0001, *n* = 6 mice/group; wild-type, *F*_(1, 13)_ = 86.84, *P* < 0.0001, *n* = 7 or 8 mice) of noise-evoked escape before and after CNO injection. (I) Summarized data of the speed of noise-evoked running before and after CNO injection (*CaMKII-Cre*, *t*_(5)_ = 13.98, *P* < 0.0001; wild-type, *t*_(5)_ = 7.474, *P* = 0.0003. Paired *t* test, *n* = 6 mice/group). (J) Schematic showing protocols for optogenetic and chemogenetic experiments. (K, L) Probability of light-evoked escape behavior (K, *U* = 0, *P* = 0.0003, Mann-Whitney *U* test, *n* = 7 or 8 mice) and time spent in the opposite chamber (L, *F*_(1, 13)_ = 19.36, *P* = 0.0007, *n* = 7 or 8 mice). (M) Summarized data of the speed of light-evoked running (*F*_(1, 13)_ = 311.2, *P* < 0.0001, *n* = 7 or 8 mice) before (pre) and during (light) light simulation. The underlying data for this figure can be found in S1 Data. Values are means ± SEM (**P < 0.01; ***P < 0.001). Two-way ANOVA with Bonferroni post hoc analysis for (H), (L), and (M). AAV, adeno-associated virus; *CaMKII*, *Ca*^*2+*^*/calmodulin-dependent protein kinase II*; CNO, clozapine-N-oxide; *Cre*, *cyclization recombination*; hM4Di, human Gi-coupled M4 muscarinic receptor; hSyn, human synapsin; IC, inferior colliculus; ICx, cortex of the inferior colliculus.
(EPS)

S12 Fig Sound-evoked escape behaviors upon inactivation of the mSC→dlPAG and ACx→lPAG pathways. (A) Schematic showing protocols for viral injection and optogenetics. (B) Typical images of the mSC (left panel) expressing AAV-DIO-eNpHR3.0-eYFP and dlPAG (right panel) containing eNpHR-expressing fibers from the mSC with a track of an optical fiber. Scale bars, 100 μm. (C) Probability of noise-evoked escape behavior (*t*_(6)_ = 6.222, *P* = 0.0008, paired *t* test, *n* = 7 mice/group) in mice with optically inhibited mSC→dlPAG projection. (D) Probability of frequency upsweeps–evoked escape behavior (*t*_(6)_ = 4.26, *P* = 0.0053, paired *t* test, *n* = 7 mice/group) in mice with optically inhibited ACx→lPAG projection. The underlying data for this figure can be found in S1 Data. Values are means ± SEM (**P < 0.01; ***P < 0.001). AAV, adeno-associated virus; ACx, auditory cortex; DIO, double-floxed inverted orientation; dlPAG, dorsolateral periaqueductal gray; eNpHR, enhanced natronomonas pharaonis halorhodopsin; eYFP, enhanced yellow fluorescent protein; lPAG, lateral periaqueductal gray; mSC, medial SC.
(EPS)

S1 Video Noise-evoked escape behavior. Duration of noise is indicated by “Noise 80 dB SPL, 5 s.” SPL, sound pressure level.
(WMV)

S2 Video Noise-evoked running in a head-fixed mouse. Duration of noise is indicated by “Noise 80 dB SPL, 5 s.” SPL, sound pressure level.
(WMV)

S3 Video Optical activation of Glul^PAG^ neurons evoked the running in a head-fixed mouse. Duration of light stimulation is indicated by “Blue Light On.” Glu, glutamatergic; lPAG, lateral periaqueductal gray.
(WMV)

S4 Video Optical activation of ACx→lPAG projections evoked escape behavior. Duration of light stimulation is indicated by “Blue Light On.” ACx, auditory cortex; lPAG, lateral periaqueductal gray.
(WMV)

S5 Video Optical activation of ACx→lPAG projections evoked the running in a head-fixed mouse. Duration of light stimulation is indicated by “Blue Light On.” ACx, auditory cortex; lPAG, lateral periaqueductal gray.
(WMV)

## Abbreviations

AAV: adeno-associated virus
ACx: auditory cortex
AMPA: α-amino-3-hydroxy-5-methyl-4-isoxazolepropionic acid
AP: anterior-posterior
CaMKII: *Ca*^*2+*^*/calmodulin-dependent protein kinase II*
ChR2: channelrhodopsin-2
CIC: central nucleus of the inferior colliculus
CNO: clozapine-N-oxide
Cre: cyclization recombination
CTB: cholera toxin subunit B
DCIC: dorsal cortex of the inferior colliculus
DIO: double-floxed inverted orientation
dlPAG: dorsolateral periaqueductal gray
DNQX: 6,7-dinitroquinoxaline-2,3(1H,4H)-dione
DV: dorsoventral
ECIC: external cortex of the inferior colliculus
Ef1α: elongation factor 1α
eNpHR: enhanced natronomonas pharaonis halorhodopsin
EnvA: avian sarcoma leucosis virus envelope protein
EPSC: excitatory postsynaptic current
eYFP: enhanced yellow fluorescent protein
GABA: γ-aminobutyric acid
Gad2: *glutamic acid decarboxylase 2*
GFP: green fluorescent protein
Glu: glutamatergic
GSH: glutathione
hM4Di: human Gi-coupled M4 muscarinic receptor
hSyn: human synapsin
IC: inferior colliculus
ICx: cortex of the inferior colliculus
lPAG: lateral periaqueductal gray
MGB: medial geniculate body
ML: mediolateral
mSC: medial superior colliculus
NMDG ACSF: N-methyl-D-glucamine artificial cerebrospinal fluid
PFA: paraformaldehyde
PSTH: peristimulus histogram
RV: rabies virus
RVG: rabies virus glycoprotein
SC: superior colliculus
SPL: sound pressure level
tdTOM: tdTomato
TTX: tetrodotoxin
vlPAG: ventrolateral periaqueductal gray
TVA: the subgroup A avian leukosis virus receptor
4-AP: 4-aminopyridine
